# The potential role of trained immunity in HIV-associated atherosclerotic cardiovascular disease: mechanisms and therapeutic implications

**DOI:** 10.3389/fimmu.2026.1918068

**Published:** 2026-08-05

**Authors:** Jinchuan Shi, Rongrong Zheng, Zhongdong Zhang

**Affiliations:** Department of Infection, Affiliated Hangzhou Xixi Hospital Zhejiang Chinese Medicine University, Hangzhou, Zhejiang, China

**Keywords:** atherosclerosis cardiovascular disease, HIV, mechanisms, therapeutic implications, trained immunity

## Abstract

**Background:**

People living with HIV (PLWH) exhibit a markedly elevated risk of developing atherosclerotic cardiovascular disease (ASCVD), a phenomenon not entirely attributable to conventional risk factors, thereby indicating the existence of an immune-mediated residual risk. Trained immunity may represent a critical underlying mechanism.

**Objective:**

This review explores the potential of HIV infection-induced trained immunity, examining its underlying mechanisms and its role in the pathophysiology of ASCVD.

**Methods:**

PubMed databases were searched for articles on the association between trained immunity and HIV infection and ASCVD.

**Results:**

HIV triggers trained immunity through various mechanisms: viral proteins like Nef reprogram monocytes, microbial translocation via LPS activates the TLR4-NF-κB pathway, CMV co-infection boosts T-cell activity, and IgA-ADCP causes cross-activation. Some ART regimens (PI/INSTI) promote metabolic training, while CCR5 antagonists may counteract it. Clinical studies and the REPRIEVE trial show immune training markers (sCD14, sCD163) are linked to coronary issues and cardiovascular events, with statins unable to fully reduce monocyte/macrophage inflammation. Strategies include metabolic interventions, anti-IL-1β therapy, epigenetic drugs, optimizing ART, and nanobiological. Importantly, this review distinguishes between canonical innate trained immunity—driven by monocytes, macrophages, and vascular cells—and T-cell immunometabolic dysfunction, which, while coexisting in HIV infection, represents a distinct adaptive immune process. The former is our primary mechanistic focus for ASCVD.

**Conclusion:**

Trained immunity plays a pivotal role in the residual risk associated with HIV-associated ASCVD. From a mechanistic perspective, trained monocytes and macrophages contribute to foam cell formation by hindering cholesterol efflux. Concurrently, trained endothelial cells maintain vascular inflammation and enhance monocyte adhesion, collectively expediting the progression of plaque development. Elucidating the underlying regulatory mechanisms and undertaking intervention trials constitute prospective avenues for translational research.

## 1 Introduction

Human immunodeficiency virus (HIV) infection continues to represent a substantial public health challenge, with the global prevalence of people living with HIV (PLWH) estimated at 40.8 million in 2024 (1). Advances in HIV management, especially the widespread use of antiretroviral therapy (ART), have significantly reduced HIV-related illness and death, enhancing the quality of life and life expectancy for PLWH to levels similar to the general population (2, 3). PLWH life expectancy has increased, and the persistence of HIV has resulted in a progressive increase in the incidence of non-AIDS-related diseases (4). The ongoing rise of HIV-related comorbidities poses new challenges in managing PLWH, particularly with non-communicable diseases like atherosclerotic cardiovascular disease (ASCVD) now being the leading cause of death (5, 6). There is a strong link between HIV infection and ASCVD, with HIV-positive individuals facing a higher risk of hypertension, dyslipidemia, and heart attacks than those without HIV (5). HIV-related chronic inflammation and immune activation play a role in contributing to ASCVD (7). However, our understanding of the underlying regulatory mechanisms of these processes is not yet fully elucidated.

**Figure 1:**
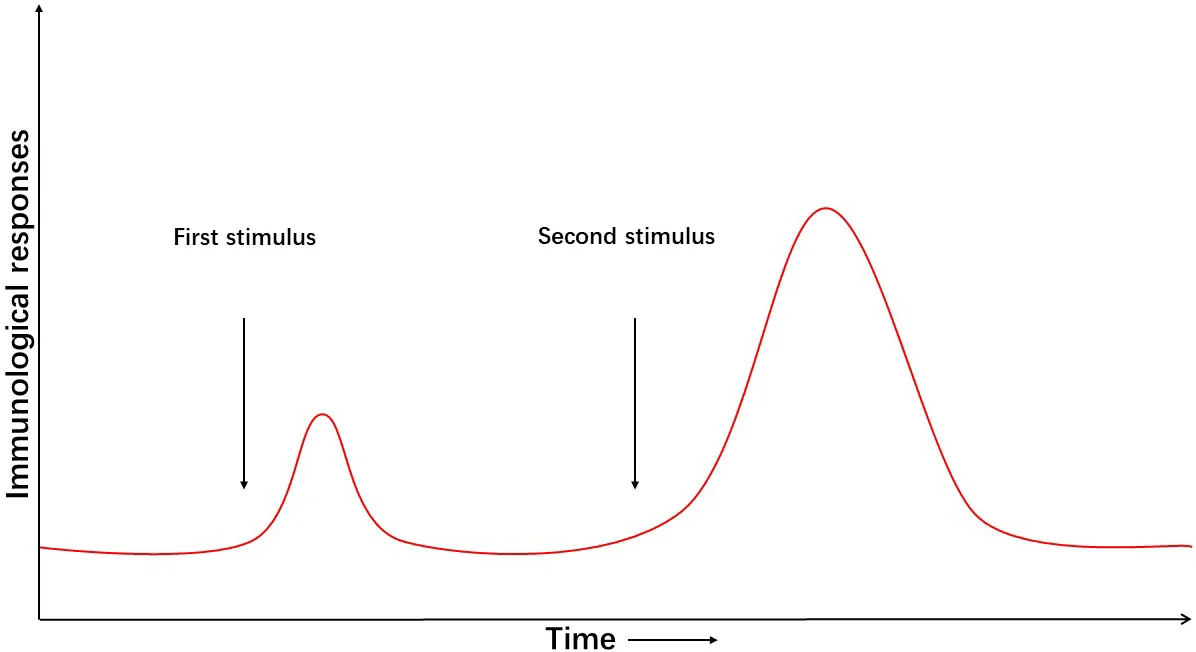
Trained immunity. Trained immunity is the process through which innate immune cells undergo long-term functional reprogramming after brief exposure to a stimulus. Reprogramming results in an augmented response after a secondary stimulus, the activation status of the immune cells returns to basal levels between the first and second stimulus, whereas their hyper-responsiveness is retained by epigenetic reprogramming.

Evidence from randomized controlled trials indicates that immunomodulatory drugs are effective in reducing cardiovascular events, thereby emphasizing the significant role of inflammation in ASCVD (8). Chronic inflammation and persistent immune activation are increasingly recognized as key contributors to HIV-associated ASCVD (5). In recent years, the concept of “trained immunity” has emerged as a novel framework for understanding the long-term memory of the innate immune system (9). Distinct from classical adaptive immunity, trained immunity describes a functional state wherein innate immune cells undergo metabolic reprogramming and epigenetic modifications following initial exposure to specific stimuli (10, 11). These alterations confer a prolonged, non-specific enhanced responsiveness, resulting in a more robust inflammatory reaction upon subsequent exposure (**Figure 1**) (10, 11). This process is critically dependent on the activation of key metabolic pathways, including glycolysis and the mevalonate pathway, as well as persistent histone modifications, particularly the trimethylation of histone H3 at lysine 4 (H3K4me3) (12).

The REPRIEVE trial indicated that elevated levels of inflammatory markers in individuals with HIV undergoing ART are a primary factor contributing to cardiovascular inflammation, implying that trained immunity significantly influence the risk of ASCVD (13). ART regimens impact cardiovascular health differently: tenofovir disoproxil fumarate (TDF)-based treatments might protect against arterial stiffness, while efavirenz (EFV) and ritonavir-boosted lopinavir (LPV/r) could harm vascular health and speed up cardiovascular disease in PLWH (14). Furthermore, co-infection with cytomegalovirus (CMV) may play a role in the pathogenesis of coronary artery disease in PLWH by exacerbating inflammatory processes (15).

In the context of HIV infection, pathogen-associated molecular patterns, such as viral RNA, Tat protein and HIV-1 Nef protein, along with persistent microbial translocation, exemplified by lipopolysaccharide (LPS), may serve as inducers of trained immunity, thereby reshaping the phenotype and function of innate immune cells (9, 16, 17). Notably, monocytes from PLWH demonstrate persistent activation, elevated production of pro-inflammatory cytokines, and altered oxidative metabolism—characteristics that significantly overlap with the hallmarks of trained immunity (18, 19). Furthermore, even in individuals undergoing ART with undetectable viremia, immune activations are observable, suggesting that trained immunity may represent a novel biological link between HIV-associated chronic inflammation and the increased risk of ASCVD (20). However, how trained immunity impacts the inflammatory profile and cardiovascular disease risk in PLWH is unclear.

Although promising insights exist, a review on trained immunity’s role in HIV-related ASCVD is missing. This article aims to: (i) outline trained immunity’s molecular mechanisms in HIV infection; (ii) examine how HIV may trigger trained immunity, leading to vascular inflammation and plaque formation; and (iii) discuss potential therapies targeting trained immunity, such as metabolic modulators, epigenetic drugs, and anti-inflammatory agents, to reduce ASCVD risk in PLWH. By combining current evidence and new ideas, this review offers a fresh immunological perspective on HIV-associated ASCVD and a foundation for developing new anti-atherosclerotic treatments.

## 2 Methodology

This narrative review aims to examine the existing literature concerning the role of trained immunity in HIV-associated ASCVD. It seeks to explore the involvement of trained immunity in HIV infection, elucidate the regulatory mechanisms through which HIV induces trained immunity, resulting in vascular inflammation and plaque formation, and discuss current and prospective interventions that clinicians might employ to mitigate the risk of ASCVD in individuals living with HIV.

The references for this review were identified through an exhaustive search of the PubMed database, covering the period from its inception to June 2026. The search employed specific terms and Boolean combinations, including: (‘trained immunity’ OR ‘innate immune memory’ OR ‘epigenetic reprogramming’ OR ‘metabolic reprogramming’ OR ‘monocyte training’) AND (‘HIV’ OR ‘PLWH’ OR ‘people living with HIV’) AND (‘atherosclerosis’ OR ‘ASCVD’ OR ‘cardiovascular disease’ OR ‘atherogenesis’) AND (‘therapeutic potential’ OR ‘immunomodulation’ OR ‘metabolic intervention’). Additional pertinent articles were identified through manual screening of the reference lists of included studies and key reviews. Articles were selected for inclusion if they were published in English and explored the intersection of trained immunity or immunometabolism with HIV infection and/or cardiovascular disease. Conference abstracts, preprints, and non-English articles were excluded from consideration. Two authors (J.S. and R.Z.) independently screened the titles and abstracts for relevance, with any discrepancies resolved through discussion with the senior author (Z.Z.). Full-text review was performed for articles meeting the preliminary inclusion criteria, and a thematic synthesis approach was used to integrate the evidence across the identified studies.

## 3 Trained immunity

### 3.1 Definition of trained immunity

In this review, the term “trained immunity” is employed in its traditional context, specifically denoting the epigenetic and metabolic reprogramming of innate immune cells—such as monocytes, macrophages, natural killer (NK) cells, dendritic cells (DCs), and hematopoietic stem/progenitor cells (HSPCs)—which leads to a prolonged, non-specific heightened responsiveness to subsequent stimuli (10, 11). This definition deliberately excludes adaptive T-cell exhaustion and metabolic dysfunction, as these are distinct processes characterized by antigen-specific memory and inhibitory receptor signaling. Although T-cell dysfunction is undeniably present in HIV infection and contributes to chronic inflammation, it does not fall under the classical definition of trained immunity. In instances where T-cell-related immunometabolic alterations are addressed within this review, they are specifically termed “T-cell immunometabolic dysfunction” to prevent any conceptual confusion. The primary mechanistic emphasis remains on innate trained immunity and its direct involvement in vascular inflammation and atherogenesis.

Trained immunity describes the process by which innate immune cells undergo long-term functional reprogramming after brief exposure to an initial stimulus, such as a pathogen or agonist, resulting in an enhanced immune response upon subsequent encounters with the same or different stimuli (10, 11). Traditionally, the innate immune system has been viewed as a nonspecific defense mechanism (21). However, research has demonstrated that the mammalian innate immune system also possesses memory-like characteristics, a concept introduced by Netea et al. in 2011 as “trained immunity” (22). During the process of trained immunity, immune cells revert to a resting state once the initial stimulus is withdrawn (23). However, their chromatin conformation, metabolic pathways, and epigenetic modifications undergo enduring remodeling (24). As a result, upon subsequent exposure to the same or isogenic stimuli, these cells demonstrate an augmented response, such as increased cytokine production (25). While trained immunity can bolster the body’s capacity to combat infections, inadequate regulation may provoke chronic or excessive inflammatory responses, potentially resulting in maladaptive pathological outcomes (26).

### 3.2 Inducers of trained immunity

Early research on trained immunity primarily examined innate immune system cells, including monocytes/macrophages, neutrophils, NK cells, innate lymphoid cells (ILC), DCs, and microglia (24). Recent studies show that non-innate immune cells, like vascular endothelial cells (VECs), vascular smooth muscle cells (VSMC), fibroblasts, and HSPCs can also produce trained immunity (27–29).

Research indicates that both exogenous and endogenous stimuli can induce trained immunity (30) Pathogens and their components, like β-glucan (β-Glu) and LPS, are key external triggers of trained immunity (31–33). Live attenuated vaccines, like Bacillus Calmette-Guérin (BCG), smallpox, measles, oral polio, and the new vaccine against tuberculosis MTBVAC, are key in trained immunity (32). The intracellular mechanisms triggering trained immunity vary among the substances mentioned, with excellent reviews available on the topic (30, 34, 35).

## 4 Mechanisms of trained immunity

The core molecular mechanism of trained immunity involves bidirectional interactions between epigenetic modifications and metabolic reprogramming, which together establish and maintain the enhanced functional state of innate immune cells (34).

### 4.1 Epigenetic mechanisms of trained immunity

Trained immunity’s regulation through epigenetic reprogramming primarily involves histone modifications and DNA methylation of innate immunity genes (36, 37). Histone modifications represent one of the most extensively investigated epigenetic mechanisms. These modifications include H3K4me1, H3K4me3, H3K27ac, and H3K9me3, which are crucial for controlling immune responses and signaling pathways (38, 39). In macrophages trained with heparin-binding hemagglutinin (HBHA), there is a persistent elevation in H3K4me3—a mark associated with transcriptional activation—within the promoter regions of several pro-inflammatory genes, including TNF-α, IL-6, and IL-1β (40). Throughout the development of the trained immunity, there is a concurrent enrichment of H3K27ac and H3K4me1 marks in the enhancer regions of various inflammation-related genes, a process referred to as “enhancer priming” (41). Caldwell et al. conducted a comprehensive review of recent evidence concerning various forms of histone regulation that impact myeloid cell behavior in the context of inflammatory processes (42). This includes an examination of histone variant co-localization, Polycomb-mediated epigenetic activation, and histone modifications associated with neutrophil NETosis (42). Future systematic investigations are necessary to elucidate the specific roles of these modifications in the regulation of memory immunity and their interactions with other modifications.

A clinical study conducted in the Netherlands demonstrates that BCG vaccination can elicit enduring alterations in DNA methylation associated with immune responses (43). These BCG-induced modifications in DNA methylation correlate with enhanced production of interferon-gamma and IL-1β, persisting for a minimum duration of three months (43). Recent research highlights that stable DNA methylation changes are crucial for maintaining a trained immune response. Liu et al. suggest that hypomethylation at pro-inflammatory loci is a key long-term epigenetic mechanism (37). After an initial trigger, pro-inflammatory genes like TNF-α and IL-6 undergo stable demethylation, creating a lasting hypomethylated state (37). This stability aligns with the enduring effects of the trained immune response. Upon re-stimulation, this hypomethylation allows for quick activation of histone marks, enhancing the innate immune response (37).

### 4.2 Metabolic pathways of trained immunity

Upon stimulation by training immunomodulators, cells undergo directed alterations in their metabolic states, facilitating the acquisition and maintenance of the functional phenotype characteristic of trained immunity (34, 44). Concurrently, these activated metabolic pathways supply essential energy (ATP) and substrates, including acetyl-CoA and α-ketoglutarate, which are crucial for ongoing epigenetic modifications and chromatin remodeling (34). The induction, maintenance, and regulation of trained immunity are contingent upon coordinated alterations across multiple metabolic pathways, notably glycolysis, oxidative phosphorylation, glutamine catabolism, and the synthesis of cholesterol and fatty acids (24, 45, 46).

Glycolysis is among the initial metabolic pathways activated during immune priming. Upon stimulation of human primary monocytes with β-Glu, BCG, or oxidized low-density lipoprotein (oxLDL), the AKT-mTOR signaling pathway is promptly activated, leading to the upregulation of glycolysis (47, 48). The activation of mTOR is essential for the establishment of trained immunity, as evidenced by the complete inhibition of this process through the application of inhibitors such as rapamycin (47, 49). Enhanced glycolysis facilitates increased glucose uptake and its conversion into pyruvate and lactate (50). Pyruvate is subsequently transformed into acetyl-CoA, which enters the mitochondrial tricarboxylic acid (TCA) cycle, generating electron carriers such as NADH and FADH_2_ for oxidative phosphorylation (51). Importantly, the intracellular accumulation of mevalonate can mediate the induction of training-induced immunity by activating the IGF1-R and mTOR-AKT-dependent glycolysis (52).

TCA cycle not only generates electron carriers essential for oxidative phosphorylation but also produces intermediate metabolites that play a direct role in epigenetic regulation (44). In macrophages with trained immunity, there is a notable accumulation of TCA cycle intermediates such as fumarate, succinate, and malate (49). Notably, fumarate acts as an inhibitor of lysine-specific demethylase 5 (KDM5), resulting in elevated levels of histone methylation and thereby establishing a connection between metabolic states and epigenetic remodeling (49). The replenishment of TCA cycle intermediates through glutaminolysis is crucial for the *in vitro* induction of immune activation in human monocytes by β-Glu and oxLDL (48, 49). In non-immune cells, pretreatment with oxLDL and trimethylamine oxide (TMAO)—synthesized in the aorta and liver—elicits a trained immune phenotype in human aortic endothelial cells (HAECs) by modulating the transcriptome and inducing metabolic reprogramming; this process involves dynamic alterations in TCA cycle intermediates (27, 53).

The cholesterol synthesis pathway is essential for adaptive immunity (49). *In vitro* studies have demonstrated that the administration of statins to monocytes inhibits β-Glu- or oxLDL-induced adaptive immunity, underscoring the pivotal role of cholesterol synthesis in this process (52). The underlying molecular mechanism involves the transport of citrate into the cytoplasm, where it is converted into acetyl-CoA and subsequently used for the synthesis of fatty acids and cholesterol (49, 51). Additionally, lysed phosphatidylcholine and oxLDL can promote the induction of trained immunity in endothelial cells (27). These findings indicate that lipid metabolites function not only as energy sources and structural components but also as critical regulatory molecules within the trained immunity signaling network.

In summary, pattern recognition receptors in innate immune and non-innate immune cells respond to microbial or endogenous stimulus, activating metabolic pathways like glycolysis and cholesterol synthesis (27). This alters histone modifications and DNA methylation, enhancing access to genes for inflammatory and metabolic proteins, with effects persisting post-stimulation (42).

## 5 Trained immunity in HIV infection

In PLWH, a “trained immune-like” phenotype of monocytes is evident even when they are undergoing ART and have achieved viral suppression (54, 55). This is evidenced by increased cytokine production (IL-1β, IL-6, TNF-α) upon TLR stimulation, which can last over a year (54). In recent years, an increasing number of researchers have paid attention to and studied trained immunity in the context of HIV infection (9). The primary determinants of HIV-associated trained immunity can be classified into two distinct categories. The first category pertains to the virus itself and its products, notably the Nef protein conveyed by exosomes and specific HIV envelope/structural proteins, which can initiate trained immunity through the TLR pathway (9, 13). The second category involves sustained stimulation against a backdrop of chronic immune activation, such as LPS and β-Glu linked to gut microbiota dysbiosis, alongside co-infections or residual viral replication, among other factors (9, 13). A recent study has demonstrated that HIV-specific IgA induces trained immunity in monocytes through antibody-dependent cellular phagocytosis (ADCP), establishing a novel antibody-dependent pathway within the HIV-associated trained immunity network that operates independently of viral proteins, such as Nef (56). Molecularly, this involves transcriptional priming of the IL-1β pathway, metabolic shifts (increased glycolysis and cholesterol biosynthesis), altered lipid raft/TLR4 signaling, and epigenetic changes like H3K4me3 and H3K27ac, along with reduced ATP-binding cassette transporter A1 (ABCA1) expression and impaired cholesterol efflux (10, 54, 55).

Collectively, these elements induce metabolic reprogramming and epigenetic remodeling in innate immune cells, thereby establishing a persistent pro-inflammatory memory phenotype. As illustrated in **Table 1**, these factors collectively elucidate the persistence of pro-inflammatory characteristics in monocytes/macrophages—such as increased levels of IL-6 and TNF-α, and compromised cholesterol efflux—even following the administration of effective ART. This observation aligns with the enduring inflammatory foundation associated with cardiovascular disease.

**Table 1:** Inducers of HIV-associated training immunity and clinical characteristics.

Categories of inducing factors	Typical examples	Primary target cells/pathways	Correlation with clinical status	References
Viral proteins (EVs)	exNef	Increased glycolysis, increased cholesterol synthesis, lipid raft enrichment, IGF1R signaling, epigenetic reprogramming	Persist even after ART cessation and are associated with persistent inflammation and the risk of comorbidities.	(9, 57)
Viral proteins (soluble/membrane)	gp120, p17/p24/gp41, Tat	TLR2/TLR4 and their downstream signaling pathways MyD88/TRIF	May be involved in priming, but gp120 is absent from the blood following ART suppression.	(9)
Microbial translocation	LPS, β-Glu	TLR ligands drive Akt-mTOR signaling, glycolysis, and cholesterol synthesis	Associated with intestinal permeability, persistent inflammation, and ASCVD risk.	(13, 58)
Background of persistent stimulation	Residual transcription/translation, co-infections	Persistent PRR stimulation and metabolic-epigenetic coupling	Maintenance of trained immunity and amplification of inflammation.	(13)
Antibody Fc signaling	IgA-ADCP	FcαRI/CD89 signaling, increased sensitivity to TLR4 restimulation	Can bridge humoral and cellular immunity and induce a protective/training-like phenotype.	(56)

EVs, extracellular vesicles; ART, antiretroviral therapy; LPS, lipopolysaccharide; β-Glu, β-glucan; ASCVD, atherosclerotic cardiovascular disease; IgA-ADCP, IgA mediated antibody-dependent cellular phagocytosis.

## 6 Trained immunity in HIV-associated ASCVD

Cardiovascular disease has emerged as a predominant cause of non-AIDS-defining mortality among individuals living with HIV (55). Numerous epidemiological studies have demonstrated that, despite the administration of effective ART, individuals with HIV are 1.5 to 2 times more likely to experience myocardial infarction, stroke, and peripheral artery disease compared to those without HIV infection (55, 59). Although traditional cardiovascular risk factors—such as smoking, hypertension, diabetes, and dyslipidemia—partially account for the heightened risk of ASCVD in this population, HIV infection itself persists as an independent risk factor even after comprehensive adjustment for these variables (60). This finding strongly indicates that the cardiovascular risk associated with HIV involves immune-mediated mechanisms that extend beyond traditional risk factors, with trained immunity as a particularly promising candidate pathway.

Prior to elaborating on the specific pathways, it is crucial to elucidate the robustness of the evidence supporting the mechanisms discussed herein. The available evidence encompasses a range: certain pathways have been directly demonstrated in cells or tissues from PLWH, constituting “direct clinical/translational evidence”; others are substantiated by *in vitro* experiments utilizing HIV proteins or patient-derived samples, categorized as “experimental evidence”; and some are inferred from classical trained immunity models (such as BCG, β-Glu, and oxLDL), proposed as plausible mechanisms in the context of HIV, and thus classified as “hypothetical/indirect evidence”. In this section, we have endeavored to specify the nature of the supporting evidence for each mechanism, enabling readers to discern between well-established findings and hypotheses that necessitate further validation within the HIV context. With this framework in mind, the subsequent sections delineate the specific pathways through which trained immunity directly promotes atherogenesis in PLWH. These mechanisms function at two interrelated levels: (i) within the vessel wall, trained macrophages and endothelial cells undergo metabolic and epigenetic reprogramming, thereby augmenting their pro-inflammatory and pro-atherogenic activities; and (ii) circulating trained monocytes, upon recruitment to the arterial intima, differentiate into macrophages with an enhanced capacity for lipid uptake and foam cell formation. Collectively, these processes create a self-perpetuating inflammatory environment that drives the initiation, progression, and destabilization of plaques.

### 6.1 Clinical validation of trained immunity in HIV-associated ASCVD

The REPRIEVE trial, the largest of its kind for preventing ASCVD in HIV-positive individuals, enrolled 7,769 participants on ART with low to moderate cardiovascular risk (13, 61). It found that pitavastatin reduced major cardiovascular events by 36%, a greater benefit than seen in the general population, implying a stronger inflammatory component in HIV-related atherosclerosis (13, 61). While statin therapy significantly reduced low-density lipoprotein cholesterol (LDL-C) and high-sensitivity C-reactive protein (hs-CRP), it did not produce significant changes in monocyte/macrophage-derived inflammatory markers, including sCD14, sCD163, IL-1β, IL-6, and caspase-1 (13). This phenomenon of “decoupling” carries dual implications: firstly, it corroborates the traditional mechanism of action of statins, which primarily involves lipid reduction and secondarily attenuates inflammation; secondly, and more critically, it indicates that monocyte/macrophage-driven inflammatory pathways persist even under effective dual therapy with statins and ART. This persistence is where the key “residual risk,” potentially mediated by trained immunity, may reside. In light of this, Obare et al. (13) explicitly identified trained immunity as a contributing factor to HIV-associated ASCVD risk in their review. Additionally, evidence from reviews indicates that monocytes in individuals infected with HIV display transcriptional and functional traits characteristic of an educated immune phenotype. This includes the upregulation of IL-6/TNF-α-mediated inflammatory signaling pathways, downregulation of ABCA1 expression, and reduced cholesterol efflux capacity, potentially facilitating foam cell formation and exacerbating plaque instability (13). Moreover, transcriptomic analyses have corroborated that the IL-1β pathway in monocytes from HIV-infected individuals is in a “pre-activated” state, aligning with the monocyte-trained immune phenotype, and this condition can persist for over a year (55).

### 6.2 Regulatory mechanisms of trained immunity in HIV-associated ASCVD

HIV has the potential to induce and expedite the development of accelerate atherosclerosis and endothelial dysfunction through various mechanisms. Researchers attribute this phenomenon, termed “trained immunity”, to the initial HIV infection, which contributes to the predisposition of HIV-infected individuals to chronic inflammation, thereby increasing their susceptibility to cardiovascular diseases and other comorbidities. These mechanisms encompass chronic inflammation, sustained immune activation due to viral replication and opportunistic infections, direct effects of the virus on adipose tissue, alterations in cholesterol metabolism, and translocation of gut microbes (62, 63). This section will systematically examine the primary inducing factors previously outlined, encompassing HIV viral proteins, microbial translocation, CMV coinfection, IgA-ADCP, ART regimens, and the elite controller phenotype. The analysis will concentrate on the mechanisms by which these factors influence various target cell types, including immune cells (such as monocytes/macrophages, T cells, and NK cells) and non-immune cells (such as endothelial and smooth muscle cells). Furthermore, it will elucidate the molecular pathways through which these factors contribute to the initiation, progression, and regulation of the pathological processes associated with cardiovascular disease.

#### 6.2.1 HIV viral proteins (Nef, Tat, gp120)

The HIV-encoded regulatory proteins Nef and Tat, along with the envelope protein gp120, can persist within viral reservoirs or be released as exosomes even under the suppression of ART (9, 64, 65). In individuals infected with HIV, the protein Nef facilitates glycolysis and cholesterol biosynthesis through the Akt-mTOR signaling pathway, while also promoting chromatin remodeling and the upregulation of genes linked to inflammation and cholesterol metabolism (13). This process contributes to the establishment of a sustained pro-inflammatory state (13). Additionally, Nef can be transferred to uninfected monocytes via exosomes (exNef), which subsequently prompts their differentiation into macrophages that release elevated levels of pro-inflammatory cytokines upon re-stimulation with LPS (55). This response is associated with chromatin modifications in pathways related to inflammation and cholesterol metabolism, indicating Nef’s potential to induce an educated immune phenotype (55). At the molecular level, the model proposed in the review posits that glycolytic intermediates induced by exNef inhibit the activity of KDM5, resulting in increased trimethylation of H3K4me3 in the promoter regions of pro-inflammatory genes and enhancing acetylation at H3K27 (13). This epigenetic landscape maintains pro-inflammatory genes in a transcriptionally active state, thereby reinforcing the memory-like characteristics of the trained immune response (13). Nef has the capacity to impair cholesterol efflux from monocytes and macrophages through the inhibition of ABCA1, consequently facilitating foam cell transformation and the progression of atherosclerotic plaques (59, 66). Collectively, the Nef-induced metabolic reprogramming, marked by increased glycolysis and cholesterol biosynthesis, directly facilitates foam cell transformation by diminishing cholesterol efflux capacity. Simultaneously, the associated epigenetic remodeling perpetuates a pro-inflammatory transcriptome, establishing a feed-forward loop that exacerbates plaque formation.

Additionally, HIV proteins, including Tat and Nef, are implicated in the pathogenesis of atherosclerosis (66). Tat enhances cytokine production in monocytes and induces the release of CCL2, thereby increasing the adhesion of monocytes and T cells to the endothelium, which can result in endothelial dysfunction (66). Concurrently, Nef promotes lymphocyte chemotaxis and macrophage activation via CD36, further exacerbating vascular inflammation (66). Furthermore, gp120 increases pro-inflammatory factors like IL-6 and IL-8 in the endothelium, enhancing adhesion molecule activity to recruit monocytes/macrophages and T cells, which sustains inflammation and speeds up atherosclerosis progression (59, 67). In the context of HIV infection, T cells are recruited to the vascular wall; however, their role is considered a concomitant adaptive response, which is distinct from the innate trained immunity that constitutes the primary focus of this review. These mechanisms collectively suggest the existence of a self-perpetuating inflammatory loop, wherein the trained immune response and viral proteins synergistically perpetuate systemic and local vascular inflammation. This process accelerates the progression of atherosclerosis and elevates the risk of cardiovascular events (13, 66).

HIV infection induces activation and functional changes in monocytes and macrophages, resulting in an exaggerated inflammatory response upon subsequent exposure to infection or stimuli (9). After the inflammatory response is activated, pro-inflammatory cytokines (IL-1β, IL-6, TNF-α, IFNγ, etc.) released by these innate immune cells may affect the expression of chemokines and adhesion molecules in endothelial cells, further promoting senescence of VECs, atherogenesis and plaque formation (68–70). Simultaneously, metabolic reprogramming related to trained immunity—characterized by enhanced glycolysis and increased cholesterol biosynthesis—may exacerbate the formation of foam cells and the progression of atherosclerotic plaques (13). Moreover, studies have revealed that trained immunity occurs not only in immune cells but also in endothelial and smooth muscle cells, which play a crucial role in the development of atherosclerosis (71). VECs and smooth muscle cells can be activated during vessel wall inflammation, contributing to the progression of atherosclerosis (72).

#### 6.2.2 Bacterial translocation (LPS and intestinal bacterial metabolites)

Recently, extensive research has focused on the gut microbiota, with many studies establishing a link between gut microbiota imbalance and the development of atherosclerosis (73). Interestingly, recent studies have identified the gut microbiota as a significant factor influencing the trained immunity of innate immune cells (74). Furthermore, HIV infection may alter the gut microbiome composition in patients, subsequently impacting the systemic inflammatory state (75). The progression of atherosclerosis in PLWH may be associated with unique gut microbiota features (76). Liu et al. described the role and mechanisms of glycolysis in diabetic atherosclerosis, such as trained immunity and gut microbiota (77). In PLWH, bacterial translocation-driven immune activation is significantly correlated with systemic inflammation and dysregulated immune cell activation, and is recognized as a fundamental mechanism contributing to residual cardiovascular risk following ART (13, 78, 79). In PLWH, circulating LPS levels are correlated with a range of cardiovascular risk factors and indicators of systemic inflammation (80, 81). Additionally, sCD14, LPS-binding protein (LBP), and other indirect markers of monocyte response to LPS are linked to the risk of future cardiovascular events (80, 81).

The persistent translocation of microbial products, including LPS, peptidoglycan, and fungal β-Glu, into the systemic circulation as a result of intestinal barrier dysfunction constitutes a fundamental mechanism driving chronic immune activation in PLWH (79). Early HIV infection has the potential to compromise the integrity of the mucosa-associated lymphoid tissue within the intestine, resulting in the depletion of CD4^+^T cells, with a pronounced reduction in Th17 cells (78, 80). This cellular depletion contributes to the downregulation of tight junction protein expression, thereby increasing intestinal permeability and facilitating the translocation of bacterial components, such as LPS, across the intestinal barrier into the systemic circulation (78, 80). LPS subsequently activates monocytes and macrophages via TLR4, which stimulates the production of pro-inflammatory cytokines, including TNF-α, IL-6, and IL-1, as well as the generation of reactive oxygen species (78, 80). These processes collectively exacerbate vascular endothelial inflammation and oxidative stress, thereby accelerating the onset and progression of atherosclerosis (78, 80). Mechanistically, LPS induces long-term reprogramming of innate immune cells through TLR4-mediated signaling pathways (82). Additionally, LPS downregulates crucial transporters involved in cholesterol efflux, such as ATP-binding cassette transporters ABCA1 and ABCG1, resulting in cholesterol accumulation within macrophages (13, 83). This accumulation facilitates foam cell transformation and contributes to the expansion of the lipid core within atherosclerotic plaques (13, 83). These observations indicate that microbiota translocation-driven trained immunity accelerates atherosclerosis progression in individuals infected with HIV by enhancing inflammatory responses, causing endothelial damage, and promoting foam cell formation.

In addition to monocytes and macrophages, recent findings suggest that endothelial cells can also develop a trained immune phenotype. In HAECs, exposure to oxidized oxLDL or TMAO triggers metabolic reprogramming, characterized by increased glycolysis and the accumulation of TCA cycle intermediates, as well as persistent histone modifications, such as H3K4me3, at pro-inflammatory gene loci (27, 53). This process of endothelial training leads to the sustained upregulation of adhesion molecules, including intercellular adhesion molecule-1 and vascular cell adhesion molecule-1, and chemokines, which facilitate monocyte recruitment and transendothelial migration—key initial events in the pathogenesis of atherosclerosis. Although direct evidence in HIV-exposed endothelial cells remains limited, the convergence of LPS-driven and viral protein-driven inflammatory signals on the vascular wall implies that endothelial trained immunity could be an overlooked contributor to HIV-associated ASCVD. In contrast, short-chain fatty acids (SCFAs)—comprising acetate, propionate, and butyrate—are vital microbial metabolites with anti-inflammatory properties that play a critical role in maintaining gut homeostasis. Among individuals living with HIV who are undergoing ART, there has been a noted depletion of gut-resident SCFA-producing bacteria, alongside reduced serum SCFA concentrations, which correlate with inflammatory markers (84). The diminished production of SCFAs by the gut microbiota has been documented to precede morbidity and mortality in PLWH, with decreased intestinal SCFA levels being linked to accelerated HIV progression (84, 85). SCFAs have been shown to exhibit anti-inflammatory properties and possess the capacity to modulate immune responses, metabolic pathways, and vascular integrity in animal models of atherosclerosis (86). The gut microbiome associated with HIV shares characteristics with microbiota profiles related to cardiovascular disease, particularly a reduced capacity for SCFA production (81). Furthermore, butyrate-producing bacteria, such as Odoribacter, have been inversely associated with carotid artery plaque in individuals infected with or at high risk for HIV (87). Nevertheless, the precise functions of SCFAs in the modulation of trained immunity within the context of HIV infection remain insufficiently investigated. Future research should aim to determine whether SCFA-producing microbiota are diminished in PLWH and assess whether their restoration—via dietary interventions, prebiotics, or probiotics—could mitigate the trained immunity-driven vascular inflammation that contributes to ASCVD.

#### 6.2.3 CMV co-infection

Clinical evidence suggests that more than 90% of PLWH are concurrently co-infected with CMV, a condition that may exacerbate the progression of cardiovascular disease via persistent immune activation (88). The phenomenon of trained immunity extends beyond myeloid cells to encompass NK cells and other immune cell types (59). Notably, CMV infection has been shown to induce trained immunity in NK cells, as demonstrated by the proliferation of NKG2C^+^CD57^+^ NK cells (89). This expansion occurs concurrently with the development of CMV-specific CD8^+^ T cells (89). Nonetheless, concerning the acceleration of atherosclerosis, the existing literature predominantly emphasizes the role of CMV in promoting the disease by augmenting inflammatory cytotoxicity via T-cell immunometabolic dysfunction, rather than considering it as the principal mechanism for trained immunity.

In PLWH, CMV-specific T-cell responses have been linked to an increase in carotid intima-media thickness, a decrease in carotid compliance, and the presence of carotid lesions (59). CMV-specific CX3CR1^+^CD4^+^ T cells have been observed to localize within the coronary artery wall, while CD57^+^CD4^+^ T cells, along with CX3CL1 and LFA-3, are detectable in atherosclerotic plaque tissue, indicating their potential role in the local immunopathological processes of the plaque (90, 91). The immune system of individuals infected with HIV is characterized by chronic inflammation and dysfunction (60). These findings suggest that T-cell responses induced by CMV may play a role in vascular inflammation. Nevertheless, this process is characteristic of adaptive immunity and is distinct from the innate trained immunity pathways examined in other sections of this review. This underlying condition may lead to a heightened response to CMV stimulation in PLWH, thereby creating a more conducive microenvironment for the dysregulation of trained immunity. The co-infection of HIV and CMV is associated with an accelerated progression of atherosclerosis, primarily through the mechanisms involving T cell immune senescence and the enhancement of vascular homing inflammation (92).

#### 6.2.4 IgA-mediated ADCP (IgA-ADCP)

Population-based research has demonstrated a correlation between elevated serum IgA levels and an increased risk of all-cause cardiovascular mortality, atherosclerosis-related mortality, and severe atherosclerosis (93). This suggests that IgA may play a role in novel inflammatory processes and influence atherosclerotic outcomes (93). Recent investigations have shown that ADCP mediated by IgA antibodies through the FcαR/CD89 signaling pathway not only effectively clears HIV-infected cells but also facilitates the differentiation of monocytes into antigen-presenting macrophages (AP-macrophages) with cross-presentation capabilities (56). This process activates HIV-specific CD8^+^ T-cell responses and induces trained immunity (56). While this pathway involves the activation of CD8^+^ T cells, its principal effect is mediated through the reprogramming of monocytes and macrophages, with the T-cell component regarded as a secondary adaptive response. In PLWH, IgA-mediated ADCP-induced priming positions monocytes/macrophages in a “hyper-responsive inflammatory state” (56). When this state is superimposed on the persistent HIV-associated inflammation, such as microbial translocation and Nef-containing exosomes, it may exacerbate arterial wall inflammation and lipid metabolism abnormalities, thereby promoting the progression of atherosclerosis (55, 57, 94).

#### 6.2.5 ART regimen

While ART drugs do not traditionally serve as inducers of trained immunity, they can modulate the body’s trained immunity status indirectly through interactions involving metabolic reprogramming and inflammatory signaling pathways. Furthermore, the immunometabolic effects associated with various drug classes exhibit considerable heterogeneity. The REPRIEVE study indicates that patients on abacavir (ABC) regimens face a heightened risk of experiencing a first cardiovascular event compared to those on TDF/tenofovir alafenamide (TDF/TAF) regimens (95). Protease inhibitors (PIs), particularly those regimens boosted with ritonavir, are significantly linked to adverse cardiometabolic outcomes, including dyslipidemia, insulin resistance, oxidative stress, and cellular senescence (80). *In vitro* studies further demonstrate that ritonavir-boosted PIs can induce oxidative stress, inflammatory responses, and senescence phenotypes in endothelial cells, with these effects being partially mitigated by statins (80). Integrase strand transfer inhibitors (INSTIs) typically exert a minimal influence on cardiovascular risk; however, they may be linked to an elevated risk of cardiovascular events during the initial stages of treatment, particularly within the first two years (80, 95). Among non-nucleoside reverse transcriptase inhibitors (NNRTIs), EFV is associated with increased lipid levels and a potential elevation in cardiovascular risk (96, 97). In contrast, newer-generation NNRTIs, such as dolutegravir and rilpivirine, exhibit improved metabolic safety profiles, with randomized studies indicating that dolutegravir/rilpivirine can enhance lipid profiles (98, 99). Overall, dyslipidemia, insulin resistance, and related metabolic disorders induced by drugs such as PIs may create a persistent pro-inflammatory microenvironment that facilitates trained immunity by promoting systemic low-grade inflammation and endothelial dysfunction (80, 100). Additionally, weight gain and glucose metabolism abnormalities associated with INSTIs and TAF may exacerbate the chronic inflammatory burden by inducing a metabolic syndrome-like phenotype, thereby indirectly sustaining trained immunity (80, 101, 102).

### 6.3 Comprehensive analysis

**Figure 2:**
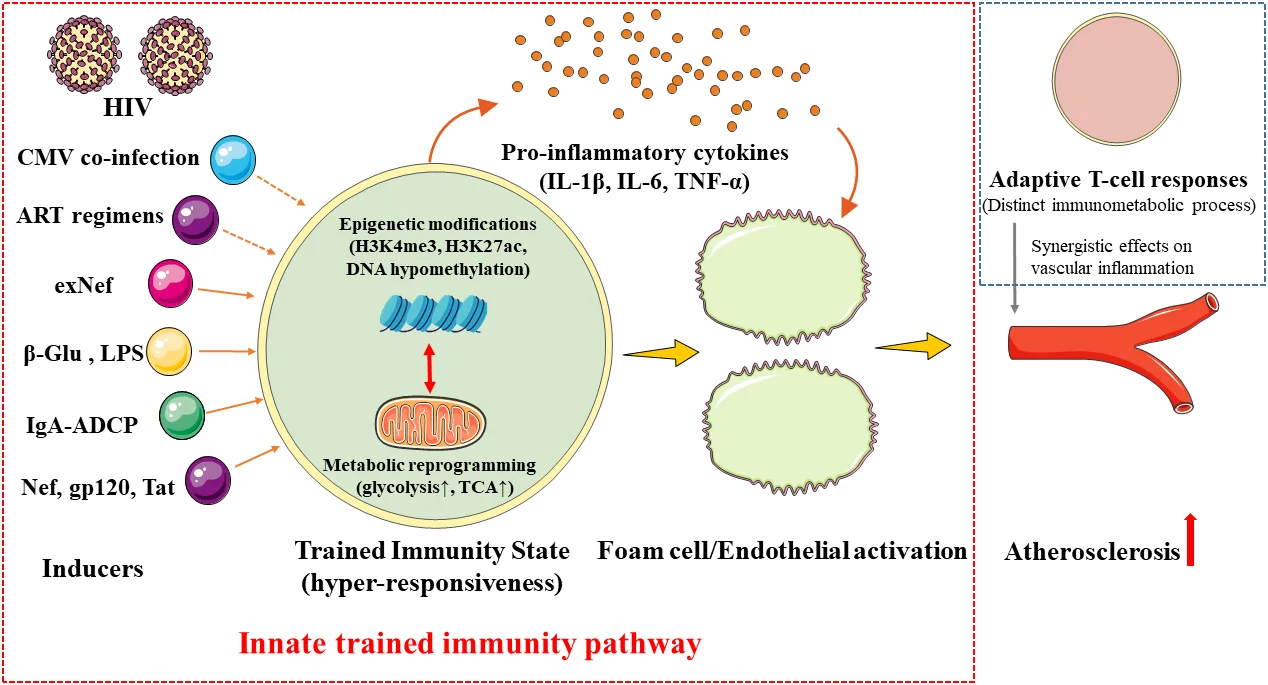
Mechanisms of trained immunity in HIV-associated atherosclerosis. HIV-derived factors—including viral proteins (Nef, gp120, Tat), exNef, microbial translocation products (LPS, β-Glu), and IgA-ADCP—act as inducers that trigger trained immunity primarily in innate immune cells and vascular wall cells. The induction of trained immunity involves a bidirectional interplay between metabolic reprogramming (enhanced glycolysis, accumulation of TCA cycle intermediates) and epigenetic modifications (H3K4me3, H3K27ac, DNA hypomethylation). This feed-forward loop sustains a long-lasting hyper-responsive state, denoted as “Trained Immunity State”. Consequently, trained innate immune cells secrete elevated levels of pro-inflammatory cytokines (IL-1β, IL-6, TNF-α), which promote foam cell formation and endothelial activation, thereby accelerating atherosclerosis. ART regimens and CMV co-infection are indicated as modulatory factors that may influence the trained immunity state. Adaptive T-cell responses are shown in a separate dashed box to indicate that they represent a distinct immunometabolic process, not central to canonical innate trained immunity. exNef, exosomal Nef; LPS, lipopolysaccharide; β-Glu, β-glucan; IgA-ADCP, IgA-mediated antibody-dependent cellular phagocytosis; TCA, tricarboxylic acid; ART, antiretroviral therapy; CMV, cytomegalovirus; H3K4me3, trimethylation of histone H3 at lysine 4; H3K27ac, acetylation of histone H3 at lysine 27.

Current evidence indicates that trained immunity significantly contributes to the development of HIV-associated ASCVD. This chapter provides a systematic review of the epidemiological and clinical trial evidence supporting the role of trained immunity as a risk factor for HIV-associated ASCVD. It further analyzes how various inducers contribute to the progression of ASCVD by influencing both immune and non-immune cells (**Table 2**, **Figure 2**). Regarding the strength of evidence, microbial translocation, particularly the LPS-sCD14 axis, and HIV viral proteins, especially Nef, are currently the most well-established immunotrophic pathways. These are supported by a comprehensive chain of evidence spanning from *in vitro* mechanisms to clinical cohort studies. In contrast, CMV coinfection and IgA-ADCP represent emerging areas of research, with their direct association with ASCVD requiring further validation. ART regimens, however, demonstrate complex bidirectional regulatory effects, highlighting the need for individualized assessment of their risk-benefit ratio. Further research is needed understand better the relationship between trained immunity and HIV-associated ASCVD and explore potential therapeutic strategies.

**Table 2:** Regulatory mechanisms of trained immunity in HIV-associated ASCVD.

Inducer	Target cells	Key mechanisms	Association with ASCVD	References
Nef	Monocytes, macrophages, lymphocytes	1. Promotes glycolysis and cholesterol biosynthesis via the Akt-mTOR pathway. 2. Inhibits ABCA1, impairs cholesterol efflux, and promotes foam cell formation. 3. Promotes macrophage activation and lymphocyte chemotaxis via CD36.	Promotes vascular inflammation, foam cell formation, and the progression of atherosclerotic plaques.	(13, 59, 66)
exNef	Uninfected monocytes → Macrophages	1. Induce differentiation into macrophages, which produce high levels of pro-inflammatory cytokines upon re-stimulation with LPS. 2. Intermediate products of glycolysis inhibit KDM5 activity → increase H3K4me3 and enhance H3K27ac → promote the transcription of pro-inflammatory genes, leading to the formation of a “trained immune” memory phenotype.	Maintaining a persistent pro-inflammatory state through epigenetic reprogramming exacerbates vascular inflammation and atherosclerosis	(13, 55)
Tat	Monocytes, T cells, endothelial cells	1. Enhances cytokine production in monocytes. 2. Induces the release of CCL2, increasing the adhesion of monocytes and T cells to the endothelium, leading to endothelial dysfunction.	Promotes endothelial dysfunction and leukocyte recruitment, and accelerates atherosclerosis.	(66)
gp120	endothelial cells	It upregulates pro-inflammatory factors such as IL-6 and IL-8, enhances the activity of adhesion molecules, and recruits monocytes/macrophages and T cells.	Maintains vascular inflammation and accelerates the progression of atherosclerosis.	(59, 67)
Trained immunity-related proinflammatory cytokines	VECs	It affects the expression of chemokines and adhesion molecules in endothelial cells, promoting the senescence of VECs.	Promotes the development of atherosclerosis and plaque formation.	(68–70)
Trained immunity-related metabolic reprogramming	Macrophages, VECs, smooth muscle cells	1. Exacerbates foam cell formation and plaque progression. 2. Is activated during inflammation of the vascular wall and contributes to the progression of atherosclerosis.	Drive metabolic inflammation and promote the onset and progression of ASCVD.	(13, 68–72)
LPS, (peptidoglycan, fungal β-Glu)	Monocytes, macrophages	1. Activates innate immune cells via TLR4, promoting the production of pro-inflammatory factors such as TNF, IL-6, and IL-1, as well as the generation of reactive oxygen species. 2. Downregulates cholesterol efflux transporters ABCA1/ABCG1 → cholesterol accumulation within macrophages → formation of foam cells. 3. Induces long-term innate immune reprogramming (trained immunity) through TLR4-mediated signaling pathways.	It exacerbates vascular endothelial inflammation and oxidative stress, promotes the formation of foam cells and the enlargement of lipid cores, and accelerates the onset and progression of atherosclerosis.	(13, 78, 80, 82, 83)
Intestinal flora imbalance + intestinal barrier dysfunction	Intestinal mucosa-associated lymphoid tissue, systemic circulation	1. Increases intestinal permeability, facilitating the translocation of bacterial products such as LPS into the systemic circulation. 2. Triggers sustained systemic immune activation, which constitutes the underlying mechanism of residual cardiovascular risk following ART.	Chronic low-grade inflammation is associated with various cardiovascular risk factors and an increased risk of future cardiovascular events (as indicated by markers such as sCD14 and LBP).	(13, 78–80)
LPS + trained immunity -related metabolic reprogramming	Macrophages, endothelial cells	Trained immunity driven by products from dysbiotic gut microbiota enhances inflammatory responses, causes endothelial damage, and promotes foam cell formation.	Accelerates the progression of atherosclerosis in PLWH.	(13, 78, 80, 83)
CMV	CMV-specific T cells (CX3CR1^+^CD4^+^ T cells, CD57^+^CD4^+^ T cells), arterial wall cells	1. CMV-specific T-cell responses are associated with increased carotid intima-media thickness, reduced carotid compliance, and carotid lesions. 2. CMV-specific T cells localize to the coronary artery wall, and associated molecules (CX3CL1, LFA-3) have been detected in atherosclerotic plaque tissue. 3. Chronic inflammation and immune dysfunction caused by HIV infection enhance the body’s response to CMV stimulation, creating a favorable microenvironment for the development of immune dysregulation.	Accelerating the progression of atherosclerosis by training the immune system.	(59, 60, 90, 91)
IgA	Monocytes → Antigen-presenting macrophages (AP-macrophages)	1. IgA-ADCP effectively clears HIV-infected cells. 2. It simultaneously promotes the differentiation of monocytes into AP-macrophages capable of cross-presentation, activates HIV-specific CD8^+^ T-cell responses, and induces memory immunity. 3. This places monocytes and macrophages in a “highly reactive inflammatory state.”	Elevated serum IgA levels are associated with an increased risk of all-cause cardiovascular mortality, atherosclerotic mortality, and severe atherosclerosis; when combined with HIV-associated chronic inflammation (such as microbial translocation and Nef-containing exosomes), this exacerbates inflammation in the arterial wall and lipid metabolism abnormalities, thereby promoting the progression of atherosclerosis.	(55–57, 94)

exNef, exosomes containing Nef protein; LPS, Lipopolysaccharide; VECs, Vascular endothelial cells; ASCVD, atherosclerotic cardiovascular disease; β-Glu, β-glucan; ART, antiretroviral therapy; CMV, Cytomegalovirus; IgA-ADCP, IgA mediated antibody-dependent cellular phagocytosis.

## 7 Therapeutic implication and future directives

### 7.1 Therapeutic strategies of targeted trained immunity

Previous evidence has shown that the mechanism of trained immunity predominantly involves epigenetic modification and metabolic reprogramming, processes regulated by enzymes that are amenable to pharmacological modulation (103). Therefore, the potential molecular mechanisms underlying trained immunity promising new targets for drug development. Multiple classes of pharmacological agents have demonstrated potential in modulating the trained immunity, as evidenced by findings from preclinical investigations and early-phase clinical trials.

#### 7.1.1 Metabolic intervention therapy

The REPRIEVE study demonstrated that among HIV-infected individuals receiving ART with low traditional cardiovascular risk factors, pitavastatin was associated with a 35% reduction in the risk of atherosclerotic cardiovascular events. This clinical finding establishes a benchmark for assessing other metabolic interventions aimed at modulating trained immunity and highlights the potential role of statins as modulators of the immunometabolic pathways that contribute to residual cardiovascular risk in PLWH (104). This finding underscores the potential significance of pitavastatin in the primary prevention of HIV-associated ASCVD (104). Importantly, the extent of cardiovascular benefit observed with pitavastatin in the REPRIEVE trial, characterized by a 35% reduction in major adverse cardiovascular events, surpasses the anticipated effects attributable solely to LDL-C reduction (104). This suggests the presence of an additional anti-inflammatory mechanism, likely associated with the inhibition of trained immunity-related pathways in monocytes and macrophages. Statins are recognized as the most extensively researched immunomodulators. Research indicates that statins can inhibit β-Glu-induced monocyte activation, a mechanism linked to decreased metabolites of the mevalonate pathway—most notably mevalonic acid—resulting from the inhibition of HMG-CoA reductase. This inhibition subsequently influences the mTOR-glycolytic axis and histone modifications, such as H3K4me3 (27, 94, 105). Previous research has indicated that statins may mitigate the inflammatory amplification effect facilitated by the HIV protein Nef by decreasing the prevalence of lipid rafts, and they almost completely abolish Nef-induced TRIM *in vitro* (106). Future studies should explore whether enhanced statin therapy or combination therapy with other anti-inflammatory agents can further attenuate the residual inflammation associated with trained immunity. In individuals with HIV and metabolic syndrome, a one-year regimen of metformin treatment has been shown to significantly decelerate the progression of coronary artery calcification and enhance insulin resistance (107). This finding suggests that metformin may hold potential value in mitigating the advancement of HIV-associated atherosclerosis (107). Mechanistically, metformin exerts its effects by inhibiting the mTOR-HIF-1α pathway through the activation of AMPK, thereby obstructing the metabolic transition associated with trained immunity from oxidative phosphorylation to aerobic glycolysis (94, 105, 108). This action prevents the establishment of trained immunity induced by β-Glu or BCG and suppresses the oxLDL-induced upregulation of glycolysis in monocytes, thereby inhibiting the development of trained immunity (94, 105, 108). Nonetheless, the cardiovascular benefits of metformin in HIV-infected populations have yet to be corroborated by large-scale clinical trials. In addition to its role in mitigating the progression of coronary artery calcium, metformin is recognized for its significant cardiovascular benefits in individuals with type 2 diabetes, including a decreased risk of myocardial infarction and cardiovascular mortality (109). These effects are partially attributed to its anti-inflammatory properties, such as the reduction of high-sensitivity C-reactive protein, IL-6, and TNF-α, rather than solely to glycemic control (110). The extent to which these cardiovascular advantages apply to PLWH who have metabolic syndrome remains to be validated through large-scale prospective studies. Nonetheless, considering that metformin inhibits trained immunity at the mechanistic level by interfering with the mTOR-HIF-1α-glycolysis pathway, it emerges as a promising candidate for addressing the residual cardiovascular risk driven by trained immunity in PLWH. Additionally, while metformin suppresses trained immunity, it may also attenuate the protective trained immunity elicited by β-Glu or BCG, potentially impacting vaccine efficacy or anti-infective immunity. Therefore, the clinical application of metformin in this context necessitates careful evaluation.

The inhibition of hexokinase by 2-deoxy-D-glucose (2-DG) and the inhibition of PFKFB3 by 3-(3-pyridyl)-1-(4-pyridyl)-2-propen-1-one (3PO) have been shown to suppress oxLDL-induced trained immunity (94, 105). Notably, 3PO has demonstrated efficacy in delaying the progression of atherosclerotic lesions in murine models, as evidenced by studies (94, 105). While 3PO has shown effectiveness in slowing the progression of atherosclerotic lesions in murine models, its applicability to PLWH has yet to be investigated. Future research should evaluate whether glycolytic inhibition, through 3PO or similar agents, can mitigate vascular inflammation and enhance cardiovascular outcomes in HIV-associated ASCVD, especially as a supplementary treatment to statins. Wang et al. discussed relevant phytochemicals that can be used to treat ASCVD by modulating monocyte/macrophage trained immunity, including the agent’s wortmannin, rapamycin, 5-Aminoimidazole-4-carboxamide ribonucleoside, metformin, ascorbate, ZVAD-fmk, 2-deoxy-d-glucose, 3-(3-Pyridinyl)-1-(4-pyridinyl)-2-propen-1-one, fluvastatin, and cerulenin, which regulate metabolic reprogramming (111). Nevertheless, the efficacy and safety of these interventions in populations infected with HIV have yet to be thoroughly assessed.

It is crucial to recognize that the cardiovascular benefits of metabolic interventions in PLWH should not be exclusively interpreted through the perspective of direct immune modulation. An increasing body of evidence suggests that both the size and transcriptional activity of the HIV reservoir are intrinsically linked to subclinical atherosclerosis (112). Persistent antigenic stimulation derived from the reservoir—even at minimal levels under suppressive ART—perpetuates chronic immune activation and maintains the trained immunity phenotype in monocytes and macrophages (13, 59). Consequently, interventions aimed at reducing the HIV reservoir—by alleviating this persistent inflammatory stimulus—may indirectly mitigate the trained immunity-mediated vascular inflammation that contributes to atherogenesis (13). This conceptual framework implies that the impact of metabolic and immunomodulatory agents on reservoir size, inflammatory markers, and vascular function should be concurrently assessed in future clinical studies to comprehensively evaluate their potential in reducing residual cardiovascular risk in PLWH.

#### 7.1.2 Epigenetic drug intervention therapy

Presently, a number of histone methylation-related pharmacological agents have shown promise in modulating trained immunity within the context of ASCVD. EZH2 inhibitors, such as EPZ-015666, GSK503, and CPI-169, have been identified as modulators of trained immunity in macrophages (94). Similarly, KDM inhibitors JIB-04 and ML-324 have demonstrated the capacity to influence macrophage epigenetic programming (94). GSK-J4, a selective inhibitor of KDM6A/KDM6B, has been shown to suppress the pro-inflammatory phenotype induced by IFN-α in monocytes (94). Furthermore, the broad-spectrum methyltransferase inhibitor MTA effectively eliminates oxLDL-induced monocyte training (94). Additionally, the DNMT inhibitor lomeguatrib has been observed to modulate macrophage training (94).

Numerous drugs associated with histone acetylation have shown promise in modulating epigenetic programming. Resveratrol, functioning as a SIRT1 activator, mitigates β-Glu-induced epigenetic programming in macrophages by augmenting deacetylase activity (94). The histone acetyltransferase inhibitor epigallocatechin gallate (EGCG) inhibits β-Glu-induced epigenetic programming (94). Similarly, the bromodomain and extra-terminal inhibitor I-BET151 reduces immune responses and suppresses epigenetic programming (94). The histone deacetylase (HDAC) inhibitor ITF2357, also known as ITF, is identified alongside MTA as an epigenetic enzyme inhibitor capable of “eliminating/reversing” epigenetic programming (94). Furthermore, a review highlights that various HDAC inhibitors, including suberoylanilide hydroxamic acid (SAHA), valproic acid (VPA), and entinostat, have the capacity to modulate immune and inflammatory responses (94, 113).

Furthermore, Wang et al. discussed relevant phytochemicals that can be used to treat ASCVD by modulating monocyte/macrophage trained immunity, including the agents Ro5-3335, 5′-deoxy-5′-methylthioadenosine, resveratrol, and epigallocatechin-3-gallate, which regulate epigenetic reprogramming (111). Nonetheless, the efficacy and safety of epigenetic drugs in patients with HIV-associated ASCVD have yet to be assessed; at present, these compounds offer only a theoretical basis for targeted interventions.

#### 7.1.3 Cytokine and inflammasome therapy

The CANTOS study showed that the IL-1β inhibitor canakinumab lowers cardiovascular events without affecting lipid levels and reduces atherosclerotic inflammation in PLWH, indicating its potential for treating HIV-associated ASCVD (104). The NLRP3 inhibitor MCC950 also decreases foam cell formation in HIV-infected macrophages, supporting further clinical research (114). Colchicine, an inflammasome-related anti-inflammatory drug, has improved cardiovascular outcomes in the general population, but its effects in PLWH need more study (104). IL-6 inhibitors, IL-1β inhibitors, methotrexate, and colchicine are promising therapies to reduce cardiovascular risk in PLWH with ASCVD (13).

#### 7.1.4 Microbial translocation and intestinal barrier repair strategies

Considering that microbial translocation serves as an upstream driver of HIV-associated trained immunity, the restoration of the intestinal barrier and the reduction of LPS translocation may effectively suppress the persistent activation of trained immunity at its origin. Current evidence indicates that HIV-associated intestinal mucosal damage and microbial translocation are linked to coronary plaque burden, and that the LPS-TLR4 axis can promote both trained immunity and atherosclerotic inflammation (80, 115). Strategies aimed at repairing the barrier, such as the use of A. muciniphila, supplementation with SCFAs or dietary fiber, and probiotic extracellular vesicles (EVs), are biologically plausible approaches to inhibit the persistent activation of trained immunity at the upstream level (116–118). Nonetheless, the causal sequence of “barrier repair—suppression of trained immunity—reduction in ASCVD events” necessitates validation through prospective intervention studies in HIV-infected populations.

#### 7.1.5 Optimization of antiviral treatment regimens

For PLWH needing metabolic risk optimization, newer NNRTIs like dolavirin and rilpivirine are preferred for their better lipid profiles (119, 120). Dolavirin, in particular, has shown superior LDL-C and non-HDL-C outcomes in trials, making it a strong option for reducing cardiovascular risk (13). Maraviroc, a CCR5 antagonist, has improved cardiovascular risk markers in PLWH on ART, indicating cardiovascular benefits for certain groups (66). Its role in inflammatory processes and existing approval for HIV treatment make maraviroc a promising candidate for “targeted immune training” strategies (121).

#### 7.1.6 Nanotechnological approaches

In a series of studies, nanotechnological approaches that act directly on inflammation of blood vessel walls or lower the trained immune system has been developed (122). Nanobiologics, composed entirely of natural molecules like apolipoproteins, are structurally stable lipid nanoparticles. They specifically target myeloid cells, are biocompatible, and can be loaded with small-molecule drugs to regulate trained immunity (123). In two separate preclinical studies, extensive experiments in mice with advanced atherosclerotic lesions demonstrated that four intravenous infusions of the nanobiologics in a week rapidly and strongly reduced vessel wall inflammation (124, 125). An alternative nanobiological strategy integrates mTOR inhibitors to diminish trained immunity and inflammation systematically. These inhibitors accumulate in the bone marrow, targeting HSPCs for sustained suppression of trained immunity, and have demonstrated safety (123). Most of the knowledge about trained immunity and its underlying mechanisms comes from *in vitro* studies isolating monocytes in humans and a few *in vivo* studies in mice. Therefore, the effects of atherosclerosis need to be explored in other training immune scenarios.

#### 7.1.7 Dietary interventions

Emerging evidence indicates that diet and lifestyle changes may influence trained immunity pathways, though research in PLWH is limited. In the general population, a Mediterranean diet is linked to lower inflammation and better heart health, possibly by altering gut microbiota and increasing SCFAs that affect immune cells (81). In HIV, various studies suggest dietary interventions could impact trained immunity and cardiovascular disease, but this area is still largely exploratory.

Research suggests that intermittent high-fat diets can alter bone marrow myeloid progenitors, leading to increased inflammation and worsened atherosclerosis, while hyperglycemia and high-salt diets can induce trained immunity in mice (126). In humans, Western diets are linked to systemic inflammation, whereas traditional plant-based diets may reduce it by regulating trained immunity (126). For PLWH, Mediterranean diets, rich in fiber and polyphenols, may decrease chronic inflammation by promoting beneficial gut microbiota, strengthening the intestinal barrier, and supporting regulatory T-cell development (127). Studies in PLWH on stable ART indicate that greater adherence to a Mediterranean diet is linked to reduced D-dimer and sTNFR2 levels, suggesting better coagulation and inflammatory responses (128). Proteomic analyses show that higher adherence enhances proteins related to antiviral defense, immunity, neuroprotection, and metabolism, while lower adherence is linked to pro-inflammatory and adverse metabolic proteins (129). A pilot RCT combining a Mediterranean diet with cholesterol-lowering foods showed reductions in LDL-C and systolic blood pressure at 6 months, but these effects were not sustained at 12 months, emphasizing the challenge of maintaining adherence (130).

There are several limitations to consider: most studies have small sample sizes, short follow-up periods, and focus on surrogate endpoints rather than definitive cardiovascular outcomes. There is also a lack of direct evidence showing that the Mediterranean diet can reverse or block HIV-associated trained immunity at the metabolic-epigenetic level. Additionally, PLWH are a diverse group with varying ART regimens, metabolic issues, and gut microbiomes, which may confound the effects of dietary interventions. Larger, long-term studies are needed to confirm findings across different populations.

### 7.2 Future directives

There exists considerable heterogeneity in the intensity of immune training and its primary determinants among individuals living with HIV. This variability is influenced by factors such as genetic background, the stage of HIV infection, ART regimens, CMV co-infections, and lifestyle. Future research should incorporate multicenter, multi-omics cohort studies to stratify patients based on the predominant factors influencing immune training (e.g., LPS-driven, CMV-driven, Nef-driven, etc.) and to develop precision intervention strategies tailored to distinct subtypes. Additionally, given the variability in individual responses to lipid-lowering and anti-inflammatory therapies, subsequent research should aim to identify high-risk subgroups that are most likely to benefit from a combined “lipid-lowering + anti-inflammatory” approach, thereby facilitating personalized treatment decisions (104).

The foundation of trained immunity is rooted in the metabolic and epigenetic reprogramming of myeloid cells. Current evidence indicates that while statins may inhibit the initiation of adaptive immunity, they are less effective in reversing established epigenetic modifications. Consequently, future therapeutic approaches should aim to target critical metabolic pathways, such as the mevalonate pathway and glycolysis, or epigenetic enzymes within a specific temporal framework to achieve more sustained regulation of inflammation (131).

### 7.3 Current limitations and challenges

Several limitations within the existing body of evidence must be recognized. Firstly, the majority of mechanistic insights into trained immunity in the context of HIV infection are derived from *in vitro* studies utilizing isolated primary monocytes or macrophage cell lines, as well as from animal models; consequently, direct evidence from longitudinal human cohorts remains scarce. Secondly, there is a lack of validated biomarkers that specifically indicate the state of trained immunity, as opposed to general inflammation markers such as hs-CRP or IL-6, which impedes both clinical evaluation and the development of targeted intervention trials. Thirdly, establishing causal relationships between trained immunity and ASCVD events in PLWH is complicated by the confounding effects of traditional cardiovascular risk factors, ART regimens, coinfections, and lifestyle factors. Fourth, the immunometabolic heterogeneity among PLWH—shaped by genetic background, stage of HIV disease, duration of ART, gut microbiome composition, and coinfections (e.g., CMV)—remains inadequately characterized. Moreover, most studies have been conducted in resource-rich settings, which limits the applicability of findings to diverse populations. Lastly, the clinical significance of the trained immunity concept in the context of ASCVD risk prediction and management remains to be conclusively determined. Although mechanistic and associative data present a compelling case, there is a notable absence of prospective interventional studies that assess therapies targeting trained immunity with definitive cardiovascular endpoints. Overcoming these limitations will be crucial for the successful translation of the trained immunity concept into clinical practice.

## 8 Conclusion and prospect

As an emerging concept that connects innate immune memory with chronic inflammatory diseases, trained immunity provides a distinctive mechanistic framework for comprehending the residual risk of ASCVD in individuals living with HIV. This review systematically explores the factors that induce HIV-associated trained immunity, including viral proteins, microbial translocation, CMV coinfection, IgA-ADCP, and ART regimens. It further delves into the molecular underpinnings of trained immunity, such as metabolic reprogramming and epigenetic modifications, and examines the clinical evidence supporting its role in the promotion of atherosclerosis. Although this field remains in its nascent stages, preliminary evidence already suggests that trained immunity constitutes an independent risk factor for HIV-associated ASCVD and may represent a promising therapeutic target for mitigating residual cardiovascular risk.

Strategies aimed at modulating the trained immune system—encompassing metabolic interventions, epigenetic drugs, anti-cytokine therapies, gut barrier repair, optimization of ART regimens such as CCR5 antagonists, and nanobiological approaches—have shown promise in preclinical and early clinical studies. Future research should prioritize the establishment of biomarkers for trained immunity, assess the impact of trained immunity on HIV-associated ASCVD, elucidate the heterogeneity of these responses, and facilitate clinical translation. A more comprehensive understanding of these mechanisms could potentially unlock novel avenues for the prevention and treatment of HIV-associated ASCVD, thereby improving long-term outcomes for affected individuals.
